# Bioactive Constituents from “Triguero” Asparagus Improve the Plasma Lipid Profile and Liver Antioxidant Status in Hypercholesterolemic Rats

**DOI:** 10.3390/ijms141121227

**Published:** 2013-10-24

**Authors:** Sara Vázquez-Castilla, Rocío De la Puerta, María Dolores García Giménez, María Angeles Fernández-Arche, Rafael Guillén-Bejarano

**Affiliations:** 1 Biotechnology Department, Instituto de la Grasa (C.S.I.C.), Seville 41012, Spain; E-Mail: rguillen@cica.es; 2 Pharmacology Department, School of Pharmacy, University of Seville, Seville 41012, Spain; E-Mails: puerta@us.es (R.D.); mdgar@us.es (M.D.G.G.); arche@us.es (M.A.F.-A.)

**Keywords:** “triguero” asparagus, flavonoids, fibers, saponins, rats, diet-induced hypercholesterolemia, plasma lipids, hepatic antioxidant enzymes

## Abstract

We have previously shown that the Andalusian-cultivated *Asparagus officinalis* L. “triguero” variety produces hypocholesterolemic and hepatoprotective effects on rats. This asparagus is a rich source of phytochemicals although we hypothesized there would be some of them more involved in these functional properties. Thus, we aimed to study the effects of asparagus (500 mg/kg body weight (bw)/day) and their partially purified fractions in flavonoids (50 mg/kg bw/day), saponins (5 mg/kg bw/day) and dietary fiber (500 mg/kg bw/day) on oxidative status and on lipid profile in rats fed a cholesterol-rich diet. After 5 weeks treatment, plasma lipid values, hepatic enzyme activities and liver malondialdehyde (MDA) concentrations were measured. With the exception of the saponin fraction (SF), the administration of lyophilized asparagus (LA), fiber fraction (FF), and flavonoid fraction (FVF) to hypercholesterolemic rats produced a significant hypolipidemic effect compare to a high-cholesterol diet (HCD). In addition, the LA and FVF groups exhibited a significant increase in enzyme activity from multiple hepatic antioxidant systems including: superoxide dismutase, catalase, and gluthatione reductase/peroxidase as well as a decrease in MDA concentrations compared to HCD group. These results demonstrate that “triguero” asparagus possesses bioactive constituents, especially dietary fiber and flavonoids, that improve the plasma lipid profile and prevent hepatic oxidative damage under conditions of hypercholesterolemia.

## 1. Introduction

It is widely accepted that hypercholesterolemia, elevated low-density lipoprotein (LDL-c) cholesterol concentration and hypertriglyceridemia are major risk factors for the development of atherosclerosis and cardiovascular disease [1]. Persistent hypercholesterolemia results from an increase in the production and secretion of LDL that prolongs its period of circulation, which increases oxidative stress and leads to the oxidative modification of LDL-c to ox-LDL-c [2,3].

It is desirable to lower total plasma cholesterol through non-pharmaceutical strategies, such as consuming foods containing bioactive compounds with hypocholesterolemic effects. Many vegetables and functional food supplements have been used for the primary prevention of cardiovascular diseases. Indeed, many studies have been conducted to identify the active ingredients responsible for the lipid-lowering properties of plant foods. Thus, for example, dietary fiber and polyphenols from vegetable products, such as cocoa and peanut skins, have been found to positively affect serum lipid and lipoprotein profiles in hypercholesterolemic rats [4,5].

Cultivated “triguero” asparagus comes from the wild autochthonous asparagus (*Asparagus officinalis* L.) from Huétor-Tajar (HT) in Andalusia, Spain. It is important to point out that there are studies that have suggested that “triguero” HT asparagus could be a hybrid between cultivated diploid varieties of *Asparagus officinalis* and the wild species *A. maritimus* [6], although some other authors did not find evidence to confirm this fact [7]. This species contains bioactive constituents such as dietary fiber, polyphenols, saponins, sterols, oligosaccharides, carotenoids and amino acids, all of which may contribute to the functional properties of this vegetable. Among the compounds with antioxidant activity, asparagus contains a large amount of polyphenols, mainly flavonoids [8,9]. The flavonoid rutin constitutes 60%–80% of the total phenolic content of purple and green asparagus extracts, and rutin could be directly related to the antioxidant properties of asparagus [10]. Moreover, “triguero” asparagus is rich in dietary fiber, particularly fructans (inulin and fructooligosaccharides) and cell wall polysaccharides containing ferulic acid residues, which also provide potential health benefits [11]. The major saponin in green asparagus is protodioscin, but studies have shown that the saponin profile in this asparagus variety is distinct; they are derived from a furostan type steroidal genin having a single bond between carbons 5 and 6 of the B ring, while protodioscin has a double bond between C5 and C6 [12]. In addition, the total saponin content of this species is higher than that of other commercial hybrids [12].

Concerning the hypolipidemic effect of this vegetable, other authors have previously investigated the cholesterol-lowering properties and hepatoprotective effects of *Asparagus racemosus* and *Asparagus officinalis* byproducts also in animal models [13,14]. Moreover, in a preliminary investigation, we demonstrated that oral supplementation with 500 mg/kg body weight of “triguero” asparagus was able to prevent increases in plasma lipid concentrations and protect against the liver oxidative damage produced by a high-cholesterol diet in rats [15]. This asparagus is a rich source of phytochemicals although we hypothesized there would be some of them more involved in these functional properties. Thus, we aimed to study the effects of partially purified fractions in flavonoids, saponins and dietary fiber from the *Asparagus officinalis* L. “triguero” variety on plasma lipid contents and hepatic antioxidant enzyme activities, in an experimental model of diet-induced hypercholesterolemia in rats. This study is therefore meant to clarify the basis for interest in the consumption of the specific asparagus “triguero”, which has a unique composition compared to other commercial green asparagus.

## 2. Results

### 2.1. Major Bioactive Constituents in the Asparagus Fractions

The major bioactive constituents in the asparagus fractions are presented in Table 1. The three main active constituents were dietary fiber, flavonoids and steroidal saponins. Dietary fiber was present at high levels in both LA and FF fractions, although its content in FF was a bit higher than in LA. The content in flavonoids in LA (10 mg/g dry matter) was almost ten times higher than in FF (1.3 mg/g dry matter). The richness in flavonoids of the purified fraction (FVF) was not high; although it was ten times greater than in LA. The saponin content was approximated in both fractions (5 and 4.4 mg/g dry matter for LA and FF, respectively) and the purified fraction (SF) presented elevated concentration in steroidal saponins (800 mg/g dry matter).

### 2.2. Animal Food Intake and Weight Gain

The rats in the HCD group had a higher average food intake than the rest of the groups (*p* < 0.05), a variable which depends on the compared experimental group (Table 2). The administration of asparagus fractions produced a reduction in body weight gain but this was especially significant for LA and FVF at the end of the experimental period, (28% (*p* < 0.05) and 51% (*p* < 0.01), respectively).

### 2.3. Plasma Lipid Profile and Atherogenic Index (AI) Values

**Figure 1:**
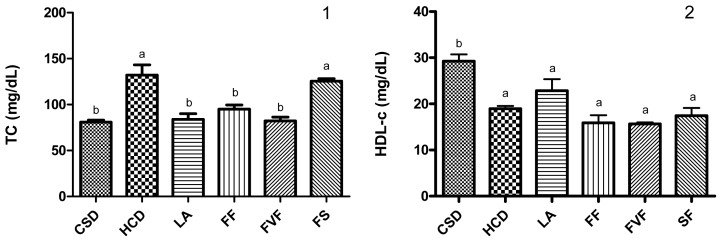

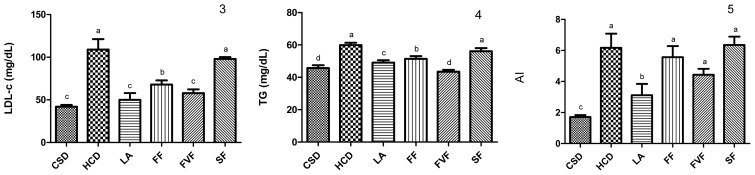
Plasma lipid concentrations from rats fed a CSD (control standard diet), HCD (high-cholesterol diet), and groups fed HCD plus LA (500 mg lyophilized asparagus/kg), FF (500 mg fiber fraction/kg), FVF (50 mg flavonoid fraction/kg) and SF (5 mg saponin fraction/kg) for five weeks. Effects on (**1**) total cholesterol (TC); (**2**) high-density lipoprotein cholesterol (HDL-c); (**3**) low-density lipoprotein cholesterol (LDL-c); (**4**) triglycerides (TG) and (**5**) atherogenic index (AI) calculated as (TC-HDLc)/HDLc. Each value represents mean ± SD from six rats. One-way ANOVA followed by Bonferroni post test. Values with different letters are significantly different from each other (*p* < 0.05).

In this experimental model, the animals fed the HCD exhibited increased plasma TG, TC, LDL-c and decreased HDL-c (*p* < 0.05) compared with the rats which were fed the control standard diet (CSD), (Figure 1). The rats of the LA, FF and FVF groups, but not of the SF group, showed significantly improved profiles (reduced TC, TG and LDL-c concentrations) in relation to the HCD group (*p* < 0.05), highlighting the LA and FVF groups, which were able to restore the lipid concentrations to the normal values of the CSD group. An increased HDL-c concentration was only observed with the LA group, although this increase was not significant. However, the calculated atherogenic index (AI) value for this group was significantly different (*p* < 0.05) from that of the HCD group.

### 2.4. Liver Antioxidant Evaluation

**Figure 2:**
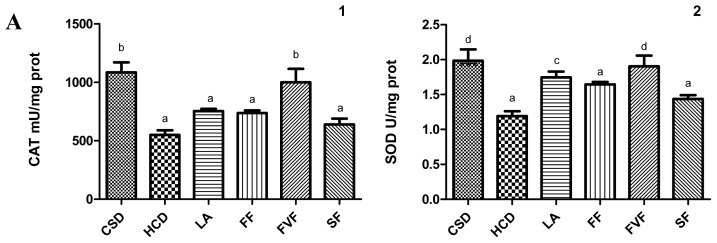

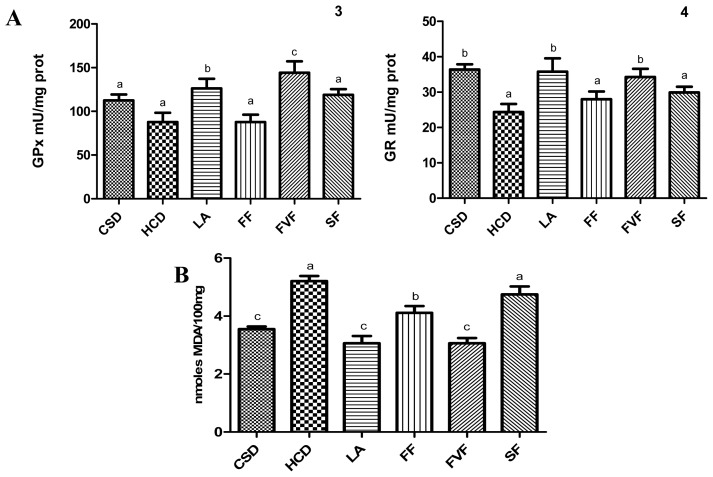
(**A**) Hepatic antioxidant enzyme activities in livers from rats fed a CSD (control standard diet), HCD (high-cholesterol diet), and groups fed HCD plus LA (500 mg lyophilized asparagus/kg), FF (500 mg fiber fraction/kg), FVF (50 mg flavonoid fraction/kg) and SF (5 mg saponin fraction/kg) for 5 weeks. Effects on (**1**) catalase (CAT); (**2**) superoxide dismutase (SOD); (**3**) glutathione peroxidase (GPx); (**4**) glutathione reductase (GR). Each value represents mean ± SD from six rats. (**B**) Malondialdehyde contents (MDA) in livers from rats fed a CSD (control standard diet), HCD (high-cholesterol diet), and groups fed HCD plus LA (500 mg lyophilized asparagus /kg), FF (500 mg fiber fraction/kg), FVF (50 mg flavonoid fraction/kg) and SF (5 mg saponin fraction/kg) for five weeks. Each value represents mean ± SD from six rats. One-way ANOVA followed by Bonferroni post test. Values with different letters are significantly different from each other (*p* < 0.05).

CAT, SOD, GPx, GR enzyme activities in livers from rats fed HCD plus lyophilized asparagus and asparagus fractions for 5 weeks compared to the CSD group are represented (Figure 2A). Oral administration of LA and FVF was able to reverse the reduced enzyme activities induced by the high-cholesterol diet. CAT activity was significantly elevated in the FVF group, and SOD, GPx, and GR activities were significantly increased in the FVF and LA groups compared to the HCD group.

The liver MDA concentrations measured in the HCD group were significantly higher than those in the CSD group (Figure 2B). The LA, FF and FVF groups, but not the SF group, showed a significant decrease (*p* < 0.05) in liver MDA concentration compared to rats fed the HCD, and this difference was greater in the LA and FVF groups.

## 3. Discussion

Rats fed a diet rich in cholesterol are often used as an experimental model for dietary hyperlipidemia; these rats exhibit increased plasma TG, TC, LDL-c, decreased circulation of HDL-c and an overweight state [16]. In our experiments, reduced food intake was observed in all the groups of animals that received asparagus samples compared to the HCD. This effect was correlated with a significant decrease in animal body weight gain in the FVF and LA groups; however, in this sense, no significant effect was found in the SF or FF groups. The protective effect produced by the asparagus administration against HCD-induced overweight is in accordance with epidemiological studies indicating that the consumption of fiber-rich vegetables prevents the development of obesity [17].

After the period of treatment, “triguero” asparagus and its major bioactive fractions, namely flavonoids and fibers, showed strong hypotriglyceridemic and hypocholesterolemic effects, reducing plasma TG, TC and LDL-c contents. In addition, increased HDL-c concentration was only observed with the LA group, and although this increase was not significant, the LA group also showed a low value of AI (*p* < 0.05) suggesting that LA might have an anti-atherogenic effect that would be beneficial for cardiovascular health. Several studies have demonstrated that high levels of HDL-c are associated with a lower incidence of cardiovascular diseases due to the inhibition of LDL oxidation and protection of endothelial cells from the cytotoxic effects of oxidized LDL [18,19].

Previous studies have demonstrated the hypolipidemic action of asparagus [13,14,20], though they did not study the biological activities of the constituents separately. From our results it can be deduced that the dietary fiber present in LA and FF at 55% and 61% respectively, might be one of the main constituents accountable for the lipid-lowering effect of the asparagus. It is well known that fiber is able to decrease plasma LDL-c concentrations by interrupting cholesterol and bile acid absorption and increasing LDL receptor activity. In fact, dietary fiber has been reported to interfere with cholesterol absorption and enterohepatic bile circulation and to cause the depletion of hepatic cholesterol pools [4,21]. In addition, our results show that, as fibers, the flavonoids from asparagus (1% dry matter) might contribute to the cholesterol-lowering effect. Flavonoids are known to down-regulate serum cholesterol content, inhibiting cholesterol synthesis and increasing the expression of LDL receptors [22]. Zhu *et al.* [14], who assayed an ethanolic and an aqueous extract from *Asparagus officinalis* by-products, discussed that the hypolipidemic effect could be due to the mixture of fiber, flavonoids and saponins. However, we could not detect modifications in any of the parameters evaluated after the administration of the saponin purified fraction (SF). It is possible that the selected dose of saponin was too low to be effective, although a dose which was not too high was deliberately chosen to avoid the known toxic effects of these phytochemicals [23]. Visavadiya and Narasimhacharya’s investigations reported that steroid-type saponins seemed to be mainly responsible for the plasma cholesterol-lowering effect in rats fed an HCD due to the reduction in the absorption of cholesterol [13]. However, these authors assayed the roots of *Asparagus racemosus* with a high steroidal saponin content (8.8% dry matter) and this is not comparable with our much smaller quantity of saponins in the LA (0.5% dry matter).

The consumption of an HCD induces damage to the rat liver due to oxidative stress caused by the excess of cholesterol in the diet. This oxidative stress damages the cells due to an increase in free radicals and consequent lipid peroxidation [24]. More recently, it has been reported that an *N*-butanol A. officinalis extract, containing 52% saponin and 2.3% flavonoids, was able to decrease the liver MDA content in mice which were fed a high-fat diet [20]. From our results, we could deduce that flavonoids seem to be the main compounds involved in this effect, since the FVF (11% in flavonoids) and the LA are the most effective fractions in preventing liver peroxidation. This finding is reasonable because it is known that flavonoids are capable of reducing lipid peroxidation due to their scavenging free radical properties [25]. The preventative peroxidation effect observed with the FF, although not highly significant, may be due to its content in certain flavonoids, and to the fact that the fiber of this asparagus variety contains other phenolics, such as ferulic acid residues in the polysaccharides of the cell wall [11].

Hepatic enzymes such as catalase, superoxide dismutase, glutathione reductase and glutathione peroxidase contribute to the antioxidant defensive mechanisms of the body. Decreases in the activities of these antioxidant enzymes were observed in hyperlipidemic rats from the HCD group, compared to those of the rats in the CSD group. However, the activities of these enzymes increased in all of the groups that received asparagus samples, especially in the animals that received the LA and the FVF fractions. Similar findings demonstrated that flavonoids were capable of stimulating the enzymatic activities of CAT and SOD [26,27]. On the other hand, our results agree findings that demonstrated that asparagus was able to increase the activities of CAT and SOD liver enzymes, restoring the antioxidant defense system [13,14].

In summary, the administration of *Asparagus officinalis* L., “triguero” variety, was shown to improve the plasma lipid profile and prevent hepatic oxidative damage under hypercholesterolemic conditions. The major bioactive constituents present in this asparagus species, such as dietary fiber and antioxidant polyphenols, especially flavonoids, seem to be highly responsible for this protective effect. These results suggest that the consumption of “triguero” asparagus, because of its composition in bioactive components, might be beneficial for cardiovascular health and thus would be considered as an effective functional food.

## 4. Experimental Section

### 4.1. Plant Extraction and Analysis

All asparagus samples were supplied by the Food Biotechnology Group of the CSIC (Seville, Spain). Asparagus spears were harvested from an experimental field in Huétor-Tájar (Andalusia, Spain). A portion of this material was lyophilized and ground to a fine powder to yield the lyophilized asparagus (LA). According to a patented method [28], another aliquot of the material was extracted with hot water and filtered; the residue was lyophilized and ground to a fine powder to yield 55% of the original dry material of a fiber purified fraction (FF) with 61% content in dietary fiber determined by gravimetric analysis [29]. The water extract was loaded into an XAD16 column previously equilibrated with water and the column was washed with 40% EtOH (4 column volumes) to yield 1.4% of the original dry material of a partially purified flavonoid fraction (FVF) with 11% content in flavonoids. Flavonoids were determined as previously described [8]. Finally, the column was washed with 90% EtOH (4 column volumes) to yield 0.5% of the original dry material of a saponin purified fraction (SF) with 80% content in saponins. Steroidal saponins were quantified using a spectrophotometric method [30].

### 4.2. Animals and Diets

Thirty-six male Wistar rats, weighing 100–125 g each, were purchased from the Central Animal House of Espartinas (Seville, Spain) and housed in an air-conditioned room at 25 ± 3 °C and 65%–70% relative humidity with a 12 h light-dark cycle. The protocol used in this study was approved by the Ethics Committee for Animal Experimentation of the University of Seville (Spain), based on the recommendations of the European Commission (2010/63/EU). The animals were randomly assigned to six groups (*n* = 6). Group CSD was fed a control standard diet (Global Diet 2014 from Harlan Laboratories, Harlan Interfauna Ibérica, S.L., Barcelona, Spain), which contains nutritional additives, including Vitamin E (120 IU/kg), Vitamin A (6000 IU/kg) and Vitamin D3 (600 IU/kg). The rest of the animals were fed a high cholesterol diet (HCD): the same control standard diet mentioned above supplemented with 1% cholesterol and 0.20% cholic acid, during 2 weeks to evoke hypercholesterolemia. After this time, the experimental groups received the HCD plus the different asparagus fractions dissolved in their drinking water during 5 weeks: 500 mg/kg body weight (bw) of the whole lyophilized asparagus (LA), 500 mg/kg bw of the fiber fraction (FF), 50 mg/kg bw of the flavonoid fraction (FVF) and 5 mg/kg bw of the saponin fraction (SF). The doses were chosen based on the knowledge of the dietary fiber, flavonoid and saponin contents present in LA and with an aim to achieve a similar or higher intake of bioactive constituents than that provided by the administration of a dose of 500 mg/kg bw of LA.

In order to monitor their intake of food and drink, the rats were housed individually in cages and were given HCD *ad libitum* and offered different dose fractions (LA, FF, FVF and SF) dissolved in 30 mL of water per day; the animals consumed approximately 95% of the prepared aqueous samples each day. The food intake was recorded daily, and the body weight was measured before the start of the experiment and every week until the end of the experiment.

At the end of the experimental period, we kept the animals fasted for 12 h, with access to pure water. The rats were anaesthetized with chloral hydrate (12% in saline), injected intraperitoneally and their blood was drawn by cardiac puncture and heparinized. To determine the plasma lipid profile, blood samples were centrifuged at 4000*g* (4 min, 4 °C) to obtain the plasma. The rats were euthanized by injecting chloral hydrate at 24% in saline. The livers were collected, weighed and rinsed with physiological saline. All samples were stored at −80 °C until analyzed.

### 4.3. Plasma Lipid Profiles

The concentrations of TC, TG and HDL-c in plasma were determined by enzymatic colorimetric methods using commercial kits (Spinreact, Spinreact, S.A., Girona, Spain). LDL-c was measured according to the procedures described by Friedewald *et al.* [31]. The atherogenic index (AI) was defined as (TC-HDL-c)/ HDL-c) and was calculated for each group.

### 4.4. Tissue Preparation and Liver Antioxidant Evaluation

Liver homogenates (10% *w*/*v*) were prepared in 0.25 M sucrose, 1 mM EDTA, 1 mM dl-dithiothreitol and 15 mM Tris-HCl (pH 7.4) and were homogenized with a Polytron homogenizer (Qiagen) at 2000 rpm using glass equipment on ice. Each homogenate was centrifuged at 800*g* for 20 min at 4 °C. The tissue protein concentration was determined according to the Bradford method [32]. The supernatant was used to determine hepatic enzyme activity.

Catalase activity (CAT) was measured using the method described by Aebi [33]. An enzymatic reaction was initiated by adding an aliquot of 20 μL of the homogenized liver and the substrate (H_2_O_2_) to a concentration of 0.5 M in a medium containing 100 mM phosphate buffer, pH 7.4. Changes in absorbance were recorded at 240 nm. CAT activity was calculated in terms of nmol H_2_O_2_ consumed/min/mg of protein and expressed in mU/mg protein.

Superoxide dismutase (SOD) activity was estimated using the xanthine-oxidase-cytochrome c method published by McCord and Fridovich [34]. This method is based on cytochrome c oxidation by superoxide anion and its dismutation by SOD. Briefly, 10 μL of the liver homogenate were mixed with potassium phosphate buffer (50 mM) (pH 7) with EDTA (0.1 mM), cytochrome c (10 μM), xanthine (50 μM), catalase (1 U) and finally xanthine-oxidase (0.04 U) which triggered the reaction. The activity was measured at 550 nm. One unit was determined as the needed enzyme to inhibit the reduction of cytochrome c by 50%. The SOD activity was expressed as U/mg of protein.

Glutathione peroxidase (GPx) activity was assayed with a coupled enzyme system in which GSSG reduction was coupled to NADPH oxidation by glutathione reductase [35]. The assay mixture contained 50 mM potassium phosphate (pH 7), 1 mM EDTA, 1 mM NaN3, 1 mM GSH, 0.2 mM NADPH, 1 U glutathione reductase and 20 μL of liver homogenate. After 5 min pre-incubation (20–25 °C), the reaction was initiated by the addition of 0.25 mM H_2_O_2_. The decrease in the absorbance at 340 nm was followed spectrophotometrically. GPx activity was calculated in terms of nmol H_2_O_2_ consumed/min/mg of protein and expressed in mU/mg of protein.

Glutathione reductase (GR) activity was determined spectrophotometrically by measuring NADPH oxidation at 340 nm [36]. The reaction mixture contained potassium phosphate (100 mM) (pH 7.6), 0.5 mM EDTA, 200 mM KCI, 0.1 mM NADPH and 100 μL of liver homogenate. After 5 min of pre-incubation at 30 °C the reaction was initiated by the addition of 1mM GSSG. GR activity was calculated in terms of nmol H_2_O_2_ consumed/min/mg of protein and expressed as mU/mg of protein.

Lipid peroxidation was estimated according to the method reported by Esterbauer and Cheeseman [37]. Briefly, 500 μL of liver homogenate was mixed with 1% (*w*/*v*) butylhydroxytoluene in acetic acid, 8% (*w*/*v*) sodiumlaurylsuphate in distilled H_2_O, 20% (*v*/*v*) acetic acid in distilled H_2_O (pH 3.4), and 0.8% (*w*/*v*) thiobarbituric acid in distilled H_2_O and then incubated for 30 min at 90 °C then cooled in ice for 5 min. After that, *N*-butanol was added, vortexed and centrifugated at 4000 rpm for 10 min. The upper organic phase was collected and the absorbance was read at 532 nm. The malondialdehyde (MDA) concentrations in each of the liver samples were determined from a standard curve generated from 1,1,3,3-tetraethoxypropane and expressed as nmol of MDA formed per 100 mg of tissue.

### 4.5. Statistic Section

The results were expressed as the mean ± standard deviations (*n* = 6). The data were evaluated by analysis of variance (ANOVA), and differences in mean values among groups were assessed using the Bonferroni post-hoc tests in a GraphPad Prism software version 5.01. *p* < 0.05 was considered to indicate a statistically significant difference.

## 5. Conclusions

These results show that cultivated *Asparagus officinalis* L. possesses bioactive constituents, especially fiber and flavonoids, that help to improve the plasma lipid profile and prevent hepatic oxidative damage under hypercholesterolemic conditions.

## Figures and Tables

**Table 1:** Major bioactive constituents (mg/g dry matter) in the asparagus fractions.

Bioactive constituents	LA	FF	FVF	SF
Dietary fiber	565 ± 14.5	610.0 ± 19.2	-	-
Flavonoids	10 ± 0.5	1.3 ± 0.1	110 ± 5.5	-
Saponins	5 ± 0.6	4.4 ± 0.4	-	800 ± 19.8

(Triplicates values: mean ± SD); LA (lyophilized asparagus), FF (fiber fraction), FVF (flavonoid fraction) and SF (saponin fraction).

**Table 2:** Weight gain and average food intake in experimental animals.

Groups	Initial body weight	Final body weight	Weight gain (g)	Average food intake (g/day)
CSD	261.5 ± 24.1	357.1 ± 29.5	95.6 ± 6.8 ^a^	12.9 ± 1.9 ^d^
HCD	252.8 ± 19.4	371.7 ± 30.1	118.9 ± 9.3 ^a^	18.7 ± 1.2 ^a^
LA	237.3 ± 17.5	322.2 ± 28.4	84.9 ± 7.4 ^b^	13.9 ± 0.9 ^c^
FF	257.3 ± 19.6	364.1 ± 34.2	106.8 ± 5.6 ^a^	13.8 ± 1.0 ^c^
FVF	245.4 ± 20.1	304.1 ± 27.6	58.7± 6.2 ^c^	15.1 ± 1.4 ^b^
SF	260.1 ± 22.3	356.1 ± 31.2	96.0 ± 8.2 ^a^	15.3 ± 1.3 ^b^

CSD (control standard diet), HCD (high-cholesterol diet), and groups fed HCD plus LA (500 mg/kg), FF (500 mg/kg), FVF (50 mg/kg) and SF (5 mg/kg) for five weeks. Each value represents mean ± standard deviation (*n* = 6). One-way ANOVA followed by Bonferroni post test. Values with different letters are significantly different from each other (*p* < 0.05).
